# Validation of Internal structure of Self-Directed Learning Readiness Scale among Indian Medical Students using factor analysis and the Structural equation Modelling Approach

**DOI:** 10.1186/s12909-021-03035-6

**Published:** 2021-12-11

**Authors:** Archana Prabu Kumar, Abirami Omprakash, Prabu Kumar Chokkalingam Mani, Narasimman Swaminathan, K. Maheshkumar, K. N. Maruthy, B. W. C. Sathiyasekaran, P. V. Vijayaraghavan, R Padmavathi

**Affiliations:** 1 Department of Physiology, Sri Ramachandra Medical College and Research Institute, Sri Ramachandra Institute of Higher Education and Research, Porur, Chennai, Tamil Nadu India; 2 Medical Education Unit, College of Medicine and Medical Sciences, Arabian Gulf University, Manama, Bahrain; 3 Department of Biochemistry, Sri Ramachandra Medical College and Research Institute, Sri Ramachandra Institute of Higher Education and Research, Porur, Chennai, Tamil Nadu India; 4 Faculty of Allied Health sciences, Sri Ramachandra Institute of Higher Education and Research, Porur, Chennai, Tamil Nadu India; 5 Department of Physiology, Government Yoga and Naturopathy Medical College and Hospital, Tamil Nadu Chennai, India; 6 Department of Physiology, Narayana Medical College, Nellore, India; 7 Department of Community Medicine, Sri Ramachandra Medical College and Research Institute, Sri Ramachandra Institute of Higher Education and Research, Porur, Chennai, Tamil Nadu India; 8 Department of Orthopaedics, Sri Ramachandra Medical College and Research Institute, Sri Ramachandra Institute of Higher Education and Research, Porur, Chennai, Tamil Nadu India

**Keywords:** Self-Directed Learning Readiness Scale (SDLRS), Medical students, Self-directed learning, Validation, Structural equation modelling (SEM)

## Abstract

**Background:**

The Self-Directed Learning Readiness Scale (SDLRS) is a tool that helps in the assessment of the readiness of the students to pursue Self-Directed Learning (SDL). There are no documented studies on the validation of internal structure of the SDLRS among Indian medical students. Hence, the objective of this study is to validate the internal structure of SDLRS among Indian medical students using factor analysis and the Structural Equation Modelling (SEM) approach.

**Methods:**

We administered Fisher’s 40-item SDLRS to 750 students after receiving the ethics clearance and the author’s permission and taking written informed consent from all the study participants (response rate: 92%). The exploratory factor analysis (EFA), confirmatory factor analysis (CFA), and Cronbach’s alpha were performed using SPSS version 25 and the Lavaan package of R version 3.1.2.

**Results:**

The values of the comparative fit index (CFI), standardised root-mean-square residual (SRMR), and root mean square error of approximation (RMSEA) were ≥ 0.9, ≤ 0.08, and ≤ 0.08, respectively, for a model fit to be acceptable. EFA showed that except for Q2 (loading score: 0.210), Q12 (loading score: 0.384), Q13 (loading score: 0.362), and Q25 (loading score: -0.219), all the items loaded well. After the exclusion of the aforementioned items, the factor loading scores for the items in the self-management, desire for learning, and self-control factors ranged from 0.405 to 0.753 (Cronbach α: 0.775), 0.396 to 0.616 (Cronbach α: 0.730), and 0.427 to 0.556 (Cronbach α: 0.799), respectively. The updated model was used for CFA, which displayed a good model fit.

**Conclusions:**

The resultant model consisting of 36 items is shown to have internal structure validity for Indian version of SDLRS, which can be used to assess medical students.

## Background

E-Learning (EL) is defined as *“Instruction delivered on a digital device that is intended to support learning”* [1]. With the rapid progress of internet technologies, EL has attracted greater attention around the world.

In the EL environment, the learners are provided with additional options to choose what to learn, when to learn and how to learn [2]. This distinctive feature of EL requires more responsibility from learners to supervise and modify their behavior to achieve the intended learning objectives [3]. Learners are expected to be responsible for their own learning, rather than waiting for teacher’s instruction or guidance [4]. The core responsibility of learning shifts from ‘teacher centered’ to ‘learner centered’ in EL [5]. In other words, the learner has to be a self-directed learner who is inclined to actively participate in all aspects of learning process such as acquisition of new knowledge, planning of activities and evaluation of the completed tasks [3].

The relevance of Self-Directed Learning (SDL) in the field of education has been emphasized since 1926 [6], even though it dates back to the era of Greek philosophers Socrates and Aristotle [7]. Houle (1961) and Tough (1971) initiated the scientific thought processes about SDL and it was Knowles who actually gave SDL a clear definition [8–10].

Knowles, described SDL as a process in which *“the students take initiate with or without the help of others, assess their learning needs, formulate goals with implementation of appropriate strategies and evaluate learning outcomes*” [10].

Efforts of several scholars to understand nuances of SDL were documented as early as 1830 [11]. Since then, many authors have added different perspectives to SDL, some of which include ‘construction of knowledge based on discussion and dialogue’, [12] ‘motivation for adults to learn’ [13], ‘promote political awareness and social action’ [14] and ‘cognitivist and constructivist approach’ [15].

Meanwhile some authors proposed different models for SDL. Long’s SDL instructional model was based on psychological control and pedagogical control with notions of four quadrants, while quadrant one is projected as an optimal option [16]. When Long concentrated on psychological and pedagogical aspects, Candy’s model recognized that students might exhibit different levels of SDL under different learning contexts [17]. Brockett and Hiemstra’s Personal Responsibility Orientation (PRO) model (1991) portrayed two dimensions of SDL, namely the process of teaching–learning and responsibility in one’s own thoughts and actions [18]. Then, Garrison proposed his model based on collaborative constructivist perspective, integrating three aspects, (1) self-management, (2) self-monitoring and (3) motivation [19]. Later Oswalt stressed on nine key concepts in his SDL model [20] .

It is stated that self-directed learners are those who feel accountable for their own learning, and are willing to explore different learning strategies, including Information and Communication Technology (ICT) [6]. SDL is believed to facilitate the trait of lifelong learning, as students with high SDL show passion to take advantage of novel opportunities like EL to learn new ability and skills [6].

Student’s readiness to pursue SDL has been shown as one of the key elements in the determination of the effectiveness of EL [21]. SDL readiness (SDLR) not only promotes student engagement in EL, but also enhance their knowledge and eventually improve their performance as well [22].

EL has been shown to enhance the possibilities of SDL significantly among students with an active internet connection [23–28]. In addition to providing students an opportunity to monitor their learning activities, EL allows understanding the contents even before attending the class, self-evaluation, continuous availability of faculty and other co-learners for interaction, which are considered as some of the mechanisms by which EL improves SDL [27]. It is documented that EL also enriches the skills of effective listening, writing, speaking, reading and comprehension by giving them endless attempts with immediate feedback to improvise [27].

The key finding, in a survey conducted among 322 online learners in US, showed that motivation was the vital element directly influencing self-monitoring behavior which indirectly altered the self-management abilities. It was proposed that, for EL to be successful, promotion of SDL skills were critical [28].

Fournier et al. (2014) described that EL can be challenging if the students lack SDLR [5]. Many authors recommend students to nurture SDLR for successfully navigating through online learning environment and obtain maximum advantage [29–31].

Given the increasing availability of ICT and EL, research on SDLR could prove quite impactful and relevant for schools and colleges [32]. Higher education institutions have been advising their students to use EL for accessing open educational resources (OER) owing to the exponential increase in knowledge, easy availability of gadgets and organizational benefits in terms of money, manpower and material [33]. With educational institutions moving towards EL in the form of flipped learning or blended learning, there is a growing need for educationists and academicians to foster SDL among students, so that learning can continue even beyond the classroom settings [33].

Robertson (2010,2011) articulates that, college students need to be empowered with suitable technical skills and tools to discover good quality learning material online, to suit their learning practices and behavior. He believes that these skills related to EL, allows them to take more control over their learning needs and encourage SDL [34, 35].

Several studies have revealed that e-courses and quizzes delivered through EL, have been shown as successful supplementary tools for enhancing motivation and SDL by promoting responsible behavior and autonomy among college students [36–39].

This trait is especially desirable in individuals pursuing the medical field because such individuals need to continually adapt to the latest research and the nuances of managing newly emerging diseases. SDLR essentially represents the extent of attitude, ability, and other traits present in a student for SDL [40]. SDL is not only an end result, but it is also an extensive process that involves taking the initiative, becoming self-dependent, identifying learning resources, implementing learning strategies, and evaluating learning outcomes [41]. Therefore, SDL is recommended for the effective and successful training of all health care professionals, including medical students, residents, doctors, and nurses [42–46].

It is a crucial educational principle that is employed by many institutes of higher education owing to its potential for developing the skills of a lifelong learner. Consequently, the Medical Council of India (MCI) has provided independent SDL hours in its newly revised curriculum to highlight the importance of SDL and ensured that all colleges follow the practice of SDL [47].

The measurement of SDLR is a prerequisite for the successful implementation of any SDL strategies. Originally, a 58-item SDLR Scale (SDLRS) was devised by Gugleilmino in 1977; it was divided into eight domains and has been used extensively for the measurement of SDL [48]. Later, in 2001, Fisher et al. introduced another SDLRS consisting of 93 items to assess the SDL among nursing students [49]. This scale was reduced to a 52-item scale by Delphi, and it was further reduced to a 40-item scale, which was divided into three domains, including ‘self-management (SM)’, ‘desire for learning (DL)’, and ’self-control (SC)’. As the number of items in the scale was reduced, its length also decreased, and there was no overall effect on its conceptual framework. Numerous studies have used the SDLRS questionnaire to evaluate the levels of SDL among medical and nursing students across many countries, including India [41, 49–56].

In India SDLRS has been used among undergraduate medical students to assess their readiness, and the resultant overall mean SDLR score was found to be 212.91, not influenced by age or gender [54]. Similar studies conducted in different parts of India, registered 140.4 ± 24.4 as mean SDLR score with male students showing higher readiness [56] and mean score of 144.6 with more readiness among female students [57].

SDLRS was also used in Indian setting, to assess the correlation between SDL and academic performance, showing higher median scores for the subscale SC (P < 0.03) among high achievers when compared to others [55]. Studies are reported from India highlighting the comparison of SDLR among medical students undergoing ‘problem-based hybrid’ curriculum with students following ‘traditional’ curriculum, showing a statistically significant increase in total median SDLR score (p = 0.004) in traditional curriculum [58].

Even though SDLRS is used extensively among medical student in India, it has not been validated in this population. This tool has been validated among different populations all over the world, except in a few countries such as India [59–65].

Williams, B (2013) suggested that SDLRS should be validated when used in newer settings [65]. In addition, to the best of our knowledge, there are no documented studies on the validation of internal structure of the SDLRS tool among Indian medical students. Therefore, this validation process is extremely essential, and this study was envisaged with the objective to develop an “Indian” version of the Fisher instrument, by validation of internal structure of the SDLRS tool among Indian medical students studying in a tertiary care medical institution by using factor analysis and the Structural Equation Modelling (SEM) approach.

## Methods

### Participants and procedures

Institutional Ethics Clearance was obtained from the host institution before the participants were recruited (ref: IEC-NI/12/OCT/30/53 dated 21.03.14). Formal permission to use the SDLRS tool was obtained from Fisher through email. Informed consent was obtained from students after providing a brief description of all the components of the study. A total of 750 first-year undergraduate medical students, comprising 250 students per batch for three consecutive academic years (2014–2017), participated in this study. Six hundred and ninety students completed the questionnaires and returned them for further analysis (response rate: 92%). To ensure uniformity, the SDLRS tool was administered for all three consecutive batches during the first month after their admission into the medical college.

Prior permission for meeting the students during one of their theory or practical classes was obtained from the concerned Heads of the Departments. Hard copies of the questionnaire were given to students, and they were given 15 to 20 min to complete the survey. The absentees during this session were followed up, and the same process was repeated until all the students had completed the questionnaire.

### Self-Directed Learning Readiness Scale Instrument

The Self-Directed Learning Readiness Scale (SDLRS) is a standardised and validated questionnaire that was developed by Murray J. Fisher [49]. The scale consists of a total of 40 items, which is a combination of three subscales: self-management (SM - 13 items), desire for learning (DL- 12 items), and self-control (SC -15 items). SDLRS has been validated by many researchers across the globe. Three-dimensional structure was supported by Fisher in 2001 and again in 2010 [49, 40] while four structured model was endorsed by Hendry and Ginns in 2009 [63]. High internal consistency for SDLRS was reported by studies from US, UK and Australia [66–68]. SDLRS by Fisher, was also validated in other languages and diverse healthcare settings [59, 69–71].

In our study students were requested to indicate the degree to which each item reflected their own characteristics using a five-point Likert scale where a score of 1 indicated strongly disagree and a score of 5 indicated strongly agree. Few questions were restructured, in order to reduce the risk of responders giving similar scores on all items without paying attention to the questions. Therefore, prior to the data analysis, those restructured items were appropriately coded (items 2, 10, 14, 18, 22, 27, 29, 36, and 37) so that higher scores for all the items indicate positive attitudes.

### Statistics

Prior to the primary analyses, duplicate, impossible, and invalid data were examined first. One case was removed due to the presence of invalid data. Next, the normality was examined using a histogram and outliers were examined using a box plot for factor analysis. No outliers were identified, and the distribution was approximately normal. Due to the large sample size, the normality and lack of outliers met the assumption for factor analysis. To cross-validate the tool, the total sample of 689 was randomly split approximately into half (N = 347 for the calibration and N = 342 for validation). First, the exploratory factor analysis (EFA) using principal component analysis was performed on the calibration sample to examine the factor structure in each of the three factors. The individual factors and the underlying items were predesignated by the previous research [49]. The goal of the EFA was to select items and improve the model. Items with factor loadings roughly, less than 0.40 was considered as a criterion for deletion from the EFA. Reliability using Cronbach’s α was used to assess the internal consistency of each factor. The scale was considered to have sufficient inter-item consistency if α > 0.70 [72]. All EFAs were analysed using SPSS version 25. Then, the confirmatory factor analysis (CFA) was conducted on the validation sample to validate the factors. The robust maximum likelihood-based estimation was used to correct for non-normality. We have assessed global goodness of fit model indices by R statistical version 4.0.2. These indices include χ2 and its subsequent ratio with degrees of freedom (χ2/df); goodness-of-fit index (GFI); comparative fit index (CFI), root mean square error of approximation (RMSEA), approximate goodness of fit indices (AGFI); normed fit index (NFI); standardized root mean square residuals (SRMR). GFI is calculated to describe how well the model fits the set of observed data and it shows the degree of variance and covariance together. The value ranges from the 0 to 1 and a value of 1 indicates a perfect fit. AGFI adjusts for the model’s degrees of freedom relative to the number of observed variables and typically range between zero and one with larger values indicating a better fit. CFI is done for comparison of null model with the fits of proposed model. If the value is greater than 0.90 means the data is acceptable. RMSEA also describes how well the model fits the observed data quantitatively. A value below 0.05 is considered as good fit. SRMR defined as closed fit and values ≤ 0.05 can be considered as a good fit and values between 0.05 and 0.08 as an adequate fit. NFI values range from 0 to 1, with higher values indicating better fit [73–75]. CFA was performed using the Lavaan package of R version 4.0.2. Finally, the frequencies and percentages were used to describe the categorical variables, mean, and standard deviation (SD) to describe each of the items, subscales, and overall scales of the SDLRS.

## Results

Data from six hundred and eighty-nine students were included in the analysis after the exclusion of one student as an invalid case. Almost all students were 18 years old, except for two students who were 17 years old. Over half of the students were females (60.7%), and most of them were Indian citizens (91.9%) (see Table 1). Both EFA and CFA were then performed to assess the validity of the tool. Descriptive statistics were done for each item, which includes mean, standard deviation, measures of skewness and kurtosis as shown in Table 2.

**Table 1:** Frequencies and percentages of demographic characteristics

Categorical variable		n	%
Gender	Male	271	39.3
	Female	418	60.7
Nationality	Indian	633	91.9
	Others	56	8.1

**Table 2:** Descriptive statistics for factors and items

	*N*	M	*SD*	Min	Max	Skewness	Kurtosis
Self-management	689	3.35	0.97	1	5	-0.04	-0.55
Q1 I manage my time well	689	3.02	1.02	1	5	-0.28	-0.12
Q3 I am organized	676	3.50	0.85	1	5	0.42	-0.81
Q4 I set strict timeframes	682	2.71	1.18	1	5	-0.07	-0.33
Q5 I have good management skills	676	3.55	0.83	1	5	0.00	-0.26
Q6 I am methodical	672	3.39	0.81	1	5	-0.07	-0.64
Q7 I am systematic in my learning	687	3.30	0.95	1	5	-0.05	-0.98
Q8 I set specific times for my study	686	3.11	1.13	1	5	-0.56	-0.08
Q9 I solve problems using a plan	668	3.46	0.97	1	5	-0.32	-0.76
Q10 I prioritize my work	677	3.47	1.05	1	5	-0.88	0.66
Q11 I can be trusted to pursue my own learning	684	3.97	0.92	1	5	-1.73	2.01
Desire for learning	689	3.95	0.93	1	5	-1.43	2.80
Q14 I want to learn new information	689	4.34	0.92	1	5	-1.02	1.37
Q15 I enjoy learning new information	683	4.21	0.87	1	5	-0.81	0.30
Q16 I have a need to learn	687	4.04	0.89	1	5	-0.59	-0.48
Q17 I enjoy a challenge	687	4.00	0.92	1	5	-0.34	0.11
Q18 I enjoy studying	688	3.75	1.11	1	5	-0.87	1.20
Q19 I critically evaluate new ideas	687	3.45	0.90	1	5	-0.56	0.41
Q20 I like to gather facts before I make a decision	688	3.92	0.86	1	5	-0.54	-0.69
Q21 I like to evaluate what I do	685	3.78	0.87	1	5	-1.32	1.86
Q22 I am open to new ideas	686	3.61	1.19	1	5	-1.11	1.93
Q23 I learn from my mistakes	684	4.18	0.92	1	5	-0.99	1.21
Q24 I need to know why	674	4.20	0.80	1	5	-0.83	-0.11
Self-control	689	3.79	0.98	1	5	-1.71	2.05
Q26 I prefer to set my own goals	683	4.07	0.86	1	5	-0.71	-0.44
Q27 I like to make decisions for myself	671	3.81	1.17	1	5	-0.40	0.07
Q28 I am responsible for my own decisions/actions	684	4.23	0.99	1	5	-0.86	1.29
Q29 I am in control of my life	687	3.77	1.18	1	5	-0.67	0.30
Q30 I have high personal standards	686	3.59	0.97	1	5	-0.62	0.58
Q31 I prefer to set my own learning goals	687	3.95	0.83	1	5	-0.89	0.85
Q32 I evaluate my own performance	684	3.74	0.90	1	5	-0.67	0.07
Q33 I am logical	685	3.81	0.86	1	5	-0.27	-0.61
Q34 I am responsible	685	3.90	0.93	1	5	-0.44	-0.82
Q35 I have high personal expectations	685	3.96	0.94	1	5	-0.54	0.07
Q36 I am able to focus on a problem	676	3.37	1.06	1	5	-0.62	-0.03
Q37 I am aware of my limitations	666	3.55	1.19	1	5	-0.54	0.39
Q38 I can find out information for myself	673	3.55	0.95	1	5	0.26	0.24
Q39 I have high beliefs in my abilities	675	3.79	1.00	1	5	-0.61	2.01
Q40 I prefer to set my own criteria on which to evaluate my performance	674	3.77	0.88	1	5	-0.27	-0.06
Total SDLR	689	137.07	14.76	99	185		

Abbreviations: SDLRS: self-directed learning readiness scale; N: number of patients; M: mean; SD: standard deviation; Min: minimum; Max: maximum

### Exploratory factor analysis

EFA was conducted on the Likert scale items for each factor of the SDLRS. According to the predefined models, the self-management factor contained 13 items (Q1–Q13), the desire for learning factor contained 12 items (Q14–Q25), and the self-control factor contained 15 items (Q26–Q40). The results of the EFA for self-management showed that three items did not load on the factor well: Q2 (“I am self-disciplined”, factor loading = 0.210), Q13 (“I am confident in my ability to search for information”, factor loading = 0.384), and Q12 (“I prefer to plan my own learning”, factor loading = 0.362). Therefore, they were sequentially trimmed from the model. The modified model on self-management comprised of Q1 and Q3–Q11, and accounted for 25.36% of the total variance. As seen in Table 3, the factor loadings for all the items ranged from 0.405 to 0.753, and the internal consistency was Cronbach α = 0.83.

**Table 3:** Exploratory factor analysis and reliability

	Factor loading	# of items	% of variance	Cronbach’s alpha
Self-management		10	25.36	0.79
Q1 I manage my time well	0.549			
Q3 I am organized	0.753			
Q4 I set strict timeframes	0.512			
Q5 I have good management skills	0.614			
Q6 I am methodical	0.488			
Q7 I am systematic in my learning	0.677			
Q8 I set specific times for my study	0.687			
Q9 I solve problems using a plan	0.486			
Q10 I prioritize my work	0.405			
Q11 I can be trusted to pursue my own learning	0.484			
Desire for learning		11	17.5	0.78
Q14 I want to learn new information	0.616			
Q15 I enjoy learning new information	0.593			
Q16 I have a need to learn	0.431			
Q17 I enjoy a challenge	0.614			
Q18 I enjoy studying	0.437			
Q19 I critically evaluate new ideas	0.435			
Q20 I like to gather facts before I make a decision	0.517			
Q21 I like to evaluate what I do	0.553			
Q22 I am open to new ideas	0.53			
Q23 I learn from my mistakes	0.396			
Q24 I need to know why	0.577			
Self-control		15	22.40	0.79
Q26 I prefer to set my own goals	0.497			
Q27 I like to make decisions for myself	0.506			
Q28 I am responsible for my own decisions/actions	0.504			
Q29 I am in control of my life	0.552			
Q30 I have high personal standards	0.542			
Q31 I prefer to set my own learning goals	0.553			
Q32 I evaluate my own performance	0.437			
Q33 I am logical	0.556			
Q34 I am responsible	0.524			
Q35 I have high personal expectations	0.44			
Q36 I am able to focus on a problem	0.454			
Q37 I am aware of my limitations	0.504			
Q38 I can find out information for myself	0.427			
Q39 I have high beliefs in my abilities	0.554			
Q40 I prefer to set my own criteria on which to evaluate my performance	0.469			
Total		36	65.29	0.83

The results of the factor analysis for desire-for-learning demonstrated that Q25 did not load on the factor well (“When presented with a problem I cannot resolve, I will ask”, factor loading = -0.219) and was poorly or inversely related to most of the other items under this factor. Therefore, Q25 was deleted from the model. The updated model on desire-for-learning explained 17.50% of the total variance and had an internal consistency of Cronbach α = 0.78. The factor loadings for Q14 through Q24 ranged from 0.396 to 0.616 (see Table 3). Although “I learn from my mistakes” had a factor loading of 0.396, it was still acceptable for inclusion in the model.

Self-control, was accounting for 22.40% of the total variance. The items from Q26 to Q40 demonstrated good inter-item reliability (Cronbach α = 0.799), and the factor loadings ranged from 0.427 to 0.556. More details on the factor loadings can be found in Table 3. Taken together, the updated models applied to the medical students comprised self-management (10 items), desire-for-learning (11 items), and self-control (15 items). All the domains of the finally modified scale showed acceptable Cronbach’s α scores, which indicated good reliability; therefore, the modified scale was used for the rest of the students (N = 342) to validate the tool.

### Confirmatory factor analyses

CFAs were conducted to verify the factor structure resulting from individual EFAs using the other half of the data (i.e., validation data). A summary for the goodness-of-fit indices from the CFA is displayed in Table 4. A summary for the goodness-of-fit indices from the CFA are displayed in Table 4. Regarding the self-management model, the fit was satisfactory (RMSEA = 0.078, SRMR = 0.056, and CFI = 0.895). Desire-for-learning model performed a good model fit (RMSEA = 0.054, SRMR = 0.052, and CFI = 0.922). Finally, self-control model was acceptable (RMSEA = 0.076, SRMR = 0.067, and CFI = 0.866). Although the CFI was a little lower than 0.900, RMSEA and SRMR indicate satisfactory model fit.

**Table 4:** CFA indices for each factor model

	RMSEA	SRMR	CFI
Self-management	0.078	0.056	0.895
Desire for learning	0.054	0.052	0.922
Self-control	0.076	0.067	0.866

Abbreviations: RMSEA: root mean square error of approximation; SRMR: standardized root-mean-square residual; CFI: comparative fit index

Lastly, the sum scores for the items under each factor were calculated from the overall dataset. Table 2 shows a summary of the descriptive statistics for each item and each factor. The average sum score for the overall SDLR was 137.07 (SD = 14.76) with a minimum score of 99 and a maximum score of 185.

### Structural equation modelling

**Fig. 1:**
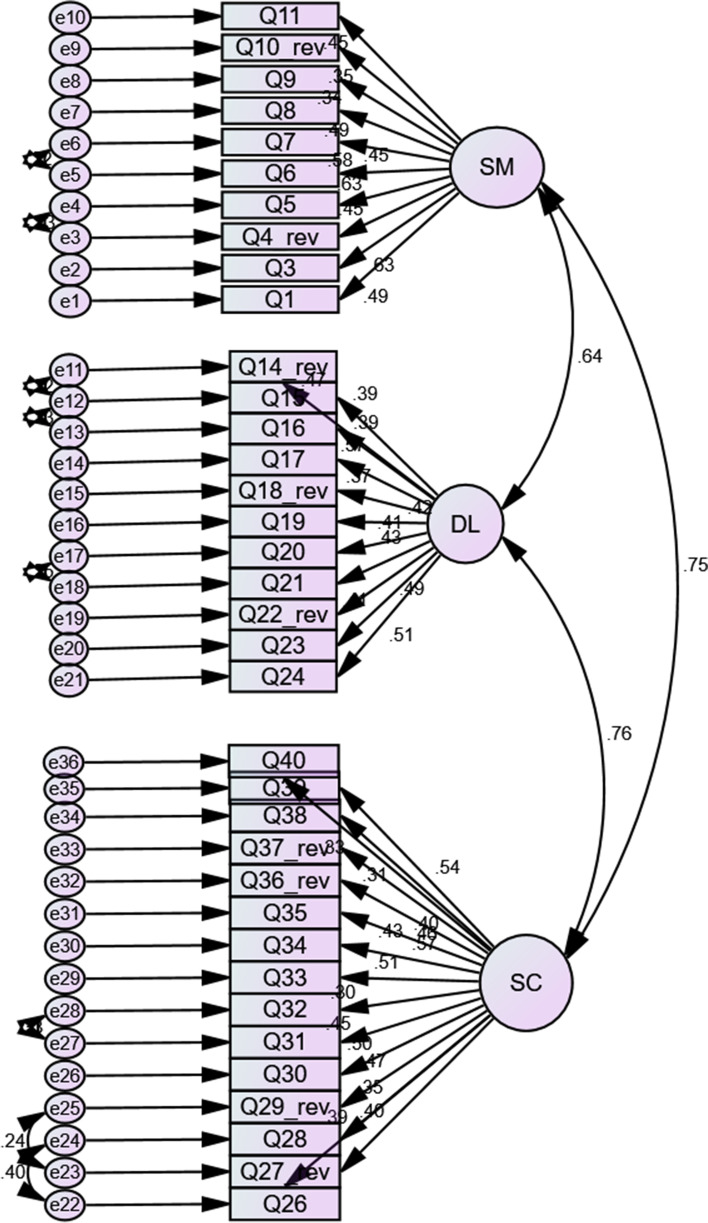
SEM results of the confirmatory factor analysis for the SDLRS model. (SM: self-management, DL: desire for learning, SC: self-control)

The CFA results were received well by ‘Analysis of a Moment Structures’ (AMOS) version 24 software, without any notification messages about the examined parameters. This shows that our factor model cleared the initial step of identification. In the subsequent step, the items (observed) and factors (unobserved) were illustrated in the hypothesised model (see Fig. 1). The factors were exemplified with rectangles; items and measurement errors were represented by ellipses and circles, respectively. The arrows running between the items and factors represented regression paths, while the numerical data shown on them indicated the standardised regression weight. The arrow between the small circles and items signified the measurement error term. The double-headed arrows extending between any two factors signified the correlation of covariance of the model. The SEM path for the SDLRS was illustrated for each item (observed) and factor (unobserved) in the hypothesised model (see Fig. 1).

## Discussion

Students’ characteristics play a vital role in determining the effectiveness of learning. This is true, especially in an online or e-learning (EL) environment, where the ability of the students to direct their efforts suitably can potentially affect the learning outcomes [3]. Medical education has witnessed substantial increase in the utilization of EL, probably due to time constraints and huge demands placed on medical students and faculty alike to find new ways to constantly update their skills and to keep track of the evolving guidelines in patient care [76, 77]. Moreover, medical students of the twenty-first century are expected to get acquainted with EL to achieve flexibility and ensure uninterrupted learning in a vibrantly changing health care setting [78]. SDL has been shown to have direct and significant influence on the cognitive processes of learners in an EL setting [3].

SDL is essential in EL because the structure of most of the e-modules are flexible and therefore needs sensible judgement from students on choosing what, when and how, they plan to engage in learning activities [2]. This flexibility necessitates students to watch their behavior and be aware that the responsibility for learning lies with them instead of the instructor or teacher [4]. Active learning approaches such as team-based learning, case based learning, flipped learning, problem-based learning, and EL have been advocated to advance the SDL competency of medical students [79, 80]. Idrosa SN et al., (2010) assessed the effect of EL on SDL among Malaysian students with the help of SDLRS and documented a significant growth in their SDL skills [81]. Interactive assignments and assessments associated with EL have been shown to enhance a sense of responsibility among leaners while kindling their excitement for exploring new learning to solve problem based activities [82–84].

A study from India has reported that downloadable EL modules enhance the opportunities for collaborative learning and SDL, as students often stored these learning resources in a pen drive and carried with them to study along with their friends, even in an offline environment [85]. Song L and Hill JR (2007) suggested number of ways by which EL nurtured SDL, such as empowering leaners to seek new information, effectively use the available resources, optimal time management, self-reflection and operational planning of goal setting [86]. In a study conducted on medical students, Svirko E et al. (2008) proposed that EL facilitates self-paced learning and self-evaluation which are the critical promotors of SDL [87].

The recognition of the attributes of SDL, especially the readiness to pursue SDL among learners permits teachers to establish guidelines on the extent of independence that can be allowed during EL. SDLRS is an instrument to measure SDL and several studies have documented across various populations that the SDLRS has high reliability and internally consistency (IC). A study among nursing students in Australia, supported the three-dimensional structure of SDLRS [49]. In a randomized experimental study conducted in UK, IC for SDLRS was reported as 0.95, with subscale scores 0.86 for ‘SM’, 0.85 for ‘DL’ and 0.89 for ‘SC’ [67]. Bridges et al., in 2007 documented IC of 0.87 for ‘SM’, 0.85 for ‘DL’ and 0.80 for ‘SC’ [66]. High IC also reported by another Australian study with Chronbach’s alpha values of 0.81 for ‘SM’, 0.78 for ‘DL’ and 0.84 for ‘SC’ [68]. These findings were found to be consistent with the results of Fisher et al. (2001) [49]. Hendry and Ginns (2009), studied the factorial structure of SDLRS among medical students through exploratory factor analysis, which revealed a four-factor model that was not in agreement with the original three-dimensional structure proposed by Fisher in 2001 [49, 63]. Fisher and Kind reexamined and confirmed the validity structure of SDLRS in 2010 [40].

SDLRS by Fisher, has been validated in other languages, including Spanish [69], Japanese [59] and Turkish [70]. SDLRS is also used in other health care professions as well, apart from nursing [55, 71, 69]. The validation of the internal structure of the SDLRS tool among Indian medical students is indispensable for measuring SDL among them. Hence, the internal structure of SDLRS tool was validated in this study.

Response rate was 92% (n = 690). A total of 36 items were included in the modified SDLRS tool because of the removal of four poorly loaded items (Q2, Q12, Q13, and Q25) as discussed under the heading of ‘results’.

The average sum score for the overall SDLR was 137.07 (SD = 14.76) with a minimum score of 99 and a maximum score of 185. This finding was comparable to a published study from India which registered total SDLR score of 132 for a hybrid medical curriculum and 137 for a traditional medical curriculum [58]. In contrast to this, another Indian study reported higher mean SDLRS scores among medical students as 144.6 (SD= 17.4), with statistically high scores among girls (p=0.002) [57]. This is probably due to the fact that the SDLR exists in all students along a continuum and is influenced by personal attributes of the learner as well as the nature of curriculum [49, 88, 89].

In our study, the mean subscale score was highest for DL (3.95±0.93), followed by SC (3.79±0.98) and SM (3.35±0.97). Our finding is comparable to a similar study conducted by Abraham et al. (2011), in different part of India, in which, the mean subscale score for DL (3.91 ± 0.20) was highest followed by SC (3.87 ± 0.16) and SM (3.44 ± 0.32) [55]. However, Devi et al., (2012) has reported maximum score for SC among Indian Medical students [58].

Our study is in agreement with other studies held in India, where SM was rated the least and authors have recommended that medical undergraduates might need additional training to improve their self-management skills [55–58].

Our study supported three structured model, which is in agreement with the findings of Fisher et al. in 2001 and 2010 [40, 49]. However, our finding do not align with study by Hendry and Ginns (2009), which revealed a four-factor model [63].

Our model with 36 items showed good fit for reliability measurement (0.83). Three factor model with ‘moderate to poor fit’ for individual subscales was revealed by Fujino-Oyama in 2016, with moderate fit for reliability measurements [59]. In an Iranian study the final model in CFA supported three factor structure with 39 items revealing a good fit of model and high internal consistency coefficients for all three factors [60]. Similar finding of high Cronbach’s alpha (>0.8) with three factor model was reported in India by Balamurugan et al., in 2015 [57].

The correlation between DL and SM was 0.64, SM and SC was calculated as 0.75 while the correlation between SC and DL was found to be 0.76 (Fig. 1).

Total variance for our model is estimated as 65.29, which is better than the original model (49.86). In our study, under the factor ‘SM’ the item ‘I set strict timeframe’ had the least mean value (2.71 ±1.18). Abraham et al. (2011) from India, also have documented least mean value for similar item, ‘I do not manage my time well’ (2.78 ± 1.20) [55] whereas Balamurugan et al., (2015) registered least value for ‘I have good management skills’ (2.7 ±1.2) [57].

For the factor DL, we recorded least value for ‘I critically evaluate new ideas’ (3.55 ± 1.00) whereas other Indian authors reported least score for item ‘I do not enjoy studying’ (3.61 ± 1.30) [55] and ‘I am open to new ideas’ (3.4 ±1.2) [57].

Lastly, under the SC factor, we registered least mean score for ‘I am able to focus on a problem’ (3.37 ± 1.06) similar to study by Abraham et.al.l (2011) with the score of 3.47 ± 1.01 [55]. However other Indian study has documented least mean score for item ‘I have high personal standards’ (2.6 ±1.1) [57]. The low mean scores could be attributed to generally perceived poor time management and critical thinking skills among medical students, as documented by other authors [90–92]. It is also known that some of the Indian medical students need guidance in time management skills. [55].

In our study the highest mean item score under SM was recorded for ‘I can be trusted to pursue my own learning’, (3.97 ± 0.92), which is in agreement with Abraham et.al.l (2011) with the score of 3.78 ±1.01 [55], and differ from other Indian author who claimed highest score for the item, ‘I am methodical’ (4.0 ± 1.0) under the same factor [57].

For the factor DL, our study showed highest item score for ‘I want to learn new information’ (4.34 ± 0.92), while other authors registered ‘I am open to new ideas’ (4.11± 0.95) [55], and ‘I like to gather the facts before I make a decision’ (4.1 ± 0.9) [57].

Finally, under SC domain the highest mean item score in our study was recorded for ‘I am responsible for my own decisions/actions’ (4.23 ± 0.99) whereas other Indian studies showed highest score for ‘I am logical’ (4.01 ± 0.83) [55], ‘I am able to focus on a problem’ ( 4.1 ± 0.9) and ‘I am not in control of my life’ ( 4.1 ± 0.9) [57].

The higher mean scores in few areas reflect the probable impact of avenues for SDL that are already being practiced in the host institution in terms of a decade-long case-based learning and enquiry-driven, integrated, and module-based curriculum. This finding is in consensus with other studies from this part of the world [56, 93]. Overall, our results indicated that the students recruited in this study were ready for SDL and support the inclusion of SDL hours in the medical curriculum.

The overall SDLR scores of the medical students of India are lower than those of the medical students from western countries, and it decreased with the increase in age [54]. On the contrary, another study conducted on nursing students showed an increase in the SDLR scores with an increase in age and maturity [94]. These results were corroborated by the results of another study conducted on Chinese nursing students [95]. Despite such contrasting results, the age and maturity of the students have been regarded as the determining factors of SDLR.

Some authors believe that it could be more challenging to promote SDL among Indian students, as most of them are trained under traditional curriculum in schools where they depend on teachers for most of their learning needs [96]. Sudesh Gyawali et al. (2011) stated that medical educators must aim to inculcate the practice of SDL among their students to promote critical thinking, which in turn would result in better information recall, retention, and eventually, better decision making. [97].

According to Vyas et al., the Integrated Learning Program (ILP) with clerkship, development of message on health education, secondary hospital program, e-learning modules, practical case discussion, early clinical exposure, topic-specific presentations by students, seminars, and assessments at end of classes play a key role in promoting SDL [54, 98, 99]. However, on the other hand, excessive curricular activities coupled with extreme socialising have been shown to lead to less time for SDL [54].

According to Shah et al. (2016), medical students generally adopt either of two types of learning: ‘surface’ learning aimed at passing the exams and ‘in-depth’ learning focusing on core concepts and their application [100]. In India, many students adopt surface learning from a young age because they are conditioned to accept the information passed down by the teacher, and due to this, they often do not understand the need for self-learning [101–103]. Measurement of SDLR among Indian medical students using a validated tool of Indian version, could help the instructors to design and modify EL accordingly.

### Limitations and directions for future research

This was a single-centre study, and to ensure uniformity in our results, we conducted the SDLR assessment among the first-year students of three consecutive batches. However, the SDLR of an individual varies according to demographic characteristics, education, and regional and cultural background. Hence, there are certain limitations on the generalisability of our results to other medical institutions in other parts of the country. Furthermore, we did not assess the SDLR of the students beyond the first year. Consequently, we could not perform any comparative analysis between the same sample set and could not determine the effect of the medical training, education, age, and maturity on the evolution of the SDLR of the students. Future studies should focus on the psychometric assessment of the SDLRS among Indian medical students across different years of study, demographics, and cultural backgrounds. In addition, future studies may also consider inclusion of qualitative data to support why some of these scores were so low and get a better idea regarding, how the students were truly operationalizing the survey items.

## Conclusions

The SDLRS is a tool that helps teachers and instructors understand the self-learning behaviour of their students, and this study is the first reported validation exercise of the internal structure of the SDLRS tool among Indian medical students. Our results have contributed to the growing body of scientific literature regarding the psychometric assessment of the SDLRS. The findings of our study did not endorse the overall construct validity of the 40-item scale; rather, they demonstrated the best model fit for the 36-item scale. This study allows future scholars and educators from India to use a more valid Indian version of the instrument in their research. Further utilisation of this validated tool as a criterion for introducing suitable changes in the digital implementation of the curriculum is recommended.

## Data Availability

The datasets generated and analysed during the current study are not publicly available due to the confidential nature of the content but are available from the corresponding author on reasonable request.

## Abbreviations

SDLRS: Self-Directed Learning Readiness Scale
SDLR: Self-Directed Learning Readiness
SDL: Self-Directed Learning
EFA: Exploratory Factor Analysis
CFA: Confirmatory Factor Analysis
SPSS: Statistical Package for the Social Sciences
CFI: Comparative Fit Index
SRMR: Standardised root-mean-square residual
RMSEA: Root mean square error of approximation
MCI: Medical Council of India
SD: standard deviation
SEM: Structural Equation Modelling
AMOS: Analysis of Moment Structures
ILP: Integrated Learning Program
